# Physiological responses and transcriptome analysis of the *Kochia prostrata* (L.) Schrad. to seedling drought stress

**DOI:** 10.3934/genet.2019.2.17

**Published:** 2019-06-05

**Authors:** Xiaojuan Wang, Jianghong Wu, Zhongren Yang, Fenglan Zhang, Hailian Sun, Xiao Qiu, Fengyan Yi, Ding Yang, Fengling Shi

**Affiliations:** 1 Inner Mongolia Agricultural University, College of Grassland, Resources and Environment, Grass resource genetic breeding, China; 2 ChiFeng University, Agricultural Science Research Institute; Grass resource genetic breeding, China; 3 Inner Mongolia Academy of Agricultural and Animal Husbandry Sciences, Inner Mongolia Grass Research Center, Chinese Academy of Sciences, Grass resource genetic breeding, China; 4 Inner Mongolia University for Nationalities, College of Animal Science and Technology, China

**Keywords:** *Kochia prostrate*, drought stress, stomatal, transcription factors, physiological changes

## Abstract

*Kochia prostrata* is a good forage plant, which has important economic and ecological value in arid and semi-arid regions of China. Drought is one of the main factors affecting its productivity. At present, there are few studies on the mechanism of drought resistance. In order to reveal the changes of physiological and biochemical indexes, stomatal structure and gene expression profiles of *Kochia prostrata* under drought treatment, the classical determination method and high-throughput Illumina Hiseq sequencing platform were applied to the control group (CK) and drought treatment group of *Kochia prostrata*. The results showed that under the condition of moderate to mild drought stress, the SOD activity reached the maximum value of 350.68 U/g min on the 5th day of stress, and under the condition of severe drought stress, the SOD activity reached the maximum on the 2nd day of stress. The accumulation of Proline remained at a high level on the 5th day of stress, and there was at least one epidermal cell interval between the two adult stomatal of the leaf epidermis, so that the evaporation shell of each stomatal did not overlap, it ensures the efficient gas exchange of the stomatal, indicating that the *Kochia prostrata* has strong drought resistance. A total of 1,177.46 M reads were obtained by sequencing, with a total of 352.25 Gbp data and Q30 of 85%. In the differential gene annotation to the biological process (BP), a total of 261 GO terms were enriched in the up-regulated genes, and a total of 231 GO terms were enriched in the down-regulated genes. The differentially expressed genes (DEGs) were obtained in 27 KEGG metabolic pathways, which laid a foundation for revealing the molecular mechanism of drought tolerance.

## 1. Introduction

Drought is a major environmental factor that limits productivity, distribution, and survivability of plants [1]. In recent years, along with industrialization urbanization and global climate change, the frequency, duration and severity of drought has increased in many regions of the world [2]–[4]. Water limitation in agriculture is poised to intensify in the coming decades. Therefore, understanding the mechanisms of drought response in plants is essential for the improvement of plant performance underwater-limiting conditions and has been the subject of many investigations over the years [5].

*Kochia prostrata* (L.) Schrad, a species of Kochia in Chenopodiaceae, is a perennial semi bushes. It is a kind of important economic forage plant in arid and semi-arid regions of China, the ecological function included such as conserving water resource preventing soil losses and fixation sand. It has lots of specialities in Ecophysiology such as high productivity, tolerance of dry, leanness-resistant tolerance, abundance of nutrition, great worth of economy and feels well [6]. It is also characterized as early turning green in spring, Strong growth and late withering in autumn, leaves are well preserved in winter. It is the main forage for livestock grazing and feeding in Sahel and desert region. It is suitable for calf and lamb fattening and dairy cow rearing. It is of great significance for the recovery of spring weak, autumn fattening and safe wintering.

Its distribution is very wide, and the soil requirements are not strict, the *Kochia prostrata* is one of the promising drought resisting species for artificial grassland and developing natural meadow. In recent years, the regulation of plant growth and development by drought stress has been deeply studied. However, there are few studies on the mechanism of resistance to *Kochia prostrata*, especially in the research of molecular drought resistance mechanism.

According to its geographical environment and the climatic characteristics of the distribution area, as well as its eco-physiological and morphological characteristics, people made a perceptual understanding of “the *Kochia prostrata* are dry and super-dry plants”. In recent years, the research group has carried out a lot of basic research and promotion work on wood mulch. The research mainly focuses on biological characteristics, nutrient composition analysis, cultivation techniques, seed coating technology, germination characteristics, stress on seed germination and seedling growth [7]–[9]. In other respects, but the research on the molecular mechanism of drought tolerance of the *Kochia prostrata* is not enough.

Polyethylene glycol (PEG) solution permeation simulates drought stress with simple and short period, and is widely used to mimic drought in many studies to investigate plant adaptive mechanisms [10]–[12]. In this study, the *Kochia prostrata* seedlings were used as materials, and the drought stress treatment group and the normal culture control group were set respectively. Under different concentrations of PEG-6000, the physiological and biochemical indexes and stomatal changes of the seedlings were compared. The transcriptome sequencing and analysis technology were used to sequence the transcript mRNA of mature leaves of the *Kochia prostrata*.

At the physiological and transcriptome levels, the mechanism of moderate drought stress on the growth and secondary metabolism was explored of the *Kochia prostrata*, its adaptability to drought stress physiological and molecular mechanisms of drought resistance were determined.

To lay the foundation for improving the quality of the *Kochia prostrata* by improving cultivation methods and biotechnology breeding techniques, providing scientific basis for further research, breeding and utilization of wild wood mulch, and the selection of returning farmland and pasture varieties in arid and semi-arid areas, The guiding significance in practice. It provides a scientific basis for the further research, breeding and utilization of wild the *Kochia prostrata*, and has a guiding significance for the selection of forage varieties in arid and semi-arid areas.

## 2. Methods

### 2.1. Plant materials and treatments

The *Kochia prostrata* seeds were immersed in a 0.5% sodium hypochlorite solution for 5 minutes for surface disinfection. The seeds with the same germination were sown in the experimental field mixed with wet sand and nutrient soil (1:1), and grown for 55 days (from the seeding count), seedling height is about 15cm for cultivation, The seedlings were placed in 1/2 Hoagland nutrient solution, and after 2 days, they were replaced with Hoagland nutrient solution, and then the nutrient solution was replaced every 3 days, and PEG-6000 (analytically pure) was used as a penetrating agent to simulate water stress. After normal growth, the seedlings were treated with Hoagland nutrient solution supplemented with PEG-6000, and Hoagland broth without PEG-6000 was used as a control.

The seedlings were planted in 45 L rectangular boxes containing different concentrations of PEG-6000 solution, 100 plants per pot, using concentration gradients of 0 (CK), 10% (mild stress), 20% (moderate stress) and 30% (Severe stress). There are 3 replicates per treatment, the first sampling between 8:30–9:00 on the first day of drought treatment, and then sampled daily for 5 days. The foil paper is wrapped quickly and frozen in liquid nitrogen, store in the refrigerator with −80 °C and take three biological replicates per treatment.

## 3. Testing index

### 3.1. Determination of plant physiological indicators

Fresh shoots (0.2 g) were thoroughly homogenized CaCO_3_ powder and 2–3 mL 5% ethanol with a mortar and pestle in the dark at 4 °C. Filter and collect the liquid, make up to 25 mL of ethanol, use 95% ethanol as blank, measure the absorbance at 665 nm, 649 nm and 470 nm using a UV-Visible spectrophotometer. Chlorophyll a (Chl a), chlorophyll b (Chl b), and total chlorophyll were estimated using the equations [13]. The free proline (Pro) content was determined by ninhydrin colorimetric method, determination of soluble sugar using anthrone method [13], determination of malondialdehyde (MDA) content by thiobarbituric acid method, Superoxide dismutase (SOD) activity was determined by nitroblue tetrazolium method (NBT), determination of peroxidase (POD) activity using guaiacol method, determination of catalase (CAT) activity using potassium permanganate titration [14].

### 3.2. Lipid peroxidation

The extent of lipid peroxidation was estimated by determining the concentration of malondialdehyde (MDA) produced by the thiobarbituric acid (TBA) reaction following the method of Draper and Hardley (1990). Shoot material (0.5 g) was homogenized in 2 mL of 0.1% (w/v) TCA solution. The homogenate was centrifuged at 15,000 g for 10 min, and 1 mL of the supernatant was added to 4 mL of 0.5% (w/v) TBA in 20% (w/v) TCA. The mixture was incubated at 90 °C for 30 min, the reaction was stopped by placing the reaction tubes in an ice water bath. Samples were centrifuged at 10,000 g for 5 min and the absorbance of the supernatant was read at 532 nm. The value for nonspecific absorption at 600 nm was subtracted. The concentration of MDA was calculated from the extinction coefficient of 155 mM^−1^ cm^−1^.

### 3.3. Stomatal structure observation

The leaves of the *Kochia prostrata* were sampled, and the stomatal structure of the leaves was observed under a Hitachi S-530 scanning electron microscope after dehydration and spray gold.

### 3.4. RNA quantification and qualification

RNA concentration was measured using NanoDrop 2000 (Thermo). RNA integrity was assessed using the RNA Nano 6000 Assay Kit of the Agilent Bioanalyzer 2100 system (Agilent Technologies, CA, USA).

### 3.5. Library preparation for transcriptome sequencing

A total amount of 1 µg RNA per sample was used as input material for the RNA sample preparations. Sequencing libraries were generated using NEBNext UltraTM RNA Library Prep Kit for Illumina (NEB, USA) following manufacturer's recommendations and index codes were added to attribute sequences to each sample. Briefly, mRNA was purified from total RNA using poly-T oligo-attached magnetic beads. Fragmentation was carried out using divalent cations under elevated temperature in NEBNext First Strand Synthesis Reaction Buffer (5X). First strand cDNA was synthesized using random hexamer primer and M-MuLV Reverse Transcriptase. Second strand cDNA synthesis was subsequently performed using DNA Polymerase I and RNase H. Remaining overhangs were converted into blunt ends via exonuclease/polymerase activities. After adenylation of 3′ ends of DNA fragments, NEBNext Adaptor with hairpin loop structure were ligated to prepare for hybridization. In order to select cDNA fragments of preferentially 240 bp in length, the library fragments were purified with AMPure XP system (Beckman Coulter, Beverly, USA). Then 3µl USER Enzyme (NEB, USA) was used with size-selected, adaptor-ligated cDNA at 37 °C for 15 min followed by 5 min at 95 °C before PCR. Then PCR was performed with Phusion High-Fidelity DNA polymerase, Universal PCR primers and Index (X) Primer. At last, PCR products were purified (AMPure XP system) and library quality was assessed on the Agilent Bioanalyzer 2100 system.

### 3.6. Clustering and sequencing

The clustering of the index-coded samples was performed on a cBot Cluster Generation System using TruSeq PE Cluster Kit v4-cBot-HS (Illumia) according to the manufacturer's instructions. After cluster generation, the library preparations were sequenced on an Illumina platform and paired-end reads were generated (The NCBI accession number of the submitted NGS data is PRJNA534334).

### 3.7. Quality control

Raw data (raw reads) of fastq format were firstly processed through in-house perl scripts. In this step, clean data (clean reads) were obtained by removing reads containing adapter, reads containing ploy-N and low quality reads from raw data. At the same time, Q20, Q30, GC-content and sequence duplication level of the clean data were calculated. All the downstream analyses were based on clean data with high quality.

### 3.8. Comparative analysis

The adaptor sequences and low-quality sequence reads were removed from the data sets. Raw sequences were transformed into clean reads after data processing. These clean reads were then mapped to the reference genome sequence. Only reads with a perfect match or one mismatch were further analyzed and annotated based on the reference genome. Hisat2 tools soft were used to map with reference genome.

### 3.9. Gene functional annotation

Gene function was annotated based on the following databases: Nr (NCBI non- redundant protein sequences); Nt (NCBI non-redundant nucleotide sequences); Swiss-Prot (A manually annotated and reviewed protein sequence database), KO (KEGG Ortholog database), GO (Gene Ontology).

### 3.10. Differential expression analysis

#### 3.10.1. GO enrichment analysis

Gene Ontology (GO) enrichment analysis of the differentially expressed genes (DEGs) was implemented by the GOseq R packages based Wallenius non-central hyper- geometric distribution [15], which can adjust for gene length bias in DEGs.

#### 3.10.2. KEGG pathway enrichment analysis

KEGG [16] is a database resource for understanding high-level functions and utilities of the biological system, such as the cell, the organism and the ecosystem, from molecular-level information, especially large-scale molecular datasets generated by genome sequencing and other high-throughput experimental technologies (http://www.genome.jp/kegg/). We used KOBAS [17] software to test the statistical enrichment of differential expression genes in KEGG pathways.

### 3.11. Statistical analysis

All the experiments were conducted with a minimum of three replicates and results were expressed as mean ± standard deviation (SD). All data were subjected to one-way analysis of variance (ANOVA) and Duncan's multiple-range test (P ≤ 0.05) using the Sigma Plot 12.0 statistical software.

## 4. Results

### 4.1. Leaf chlorophyll content

**Figure 1.**
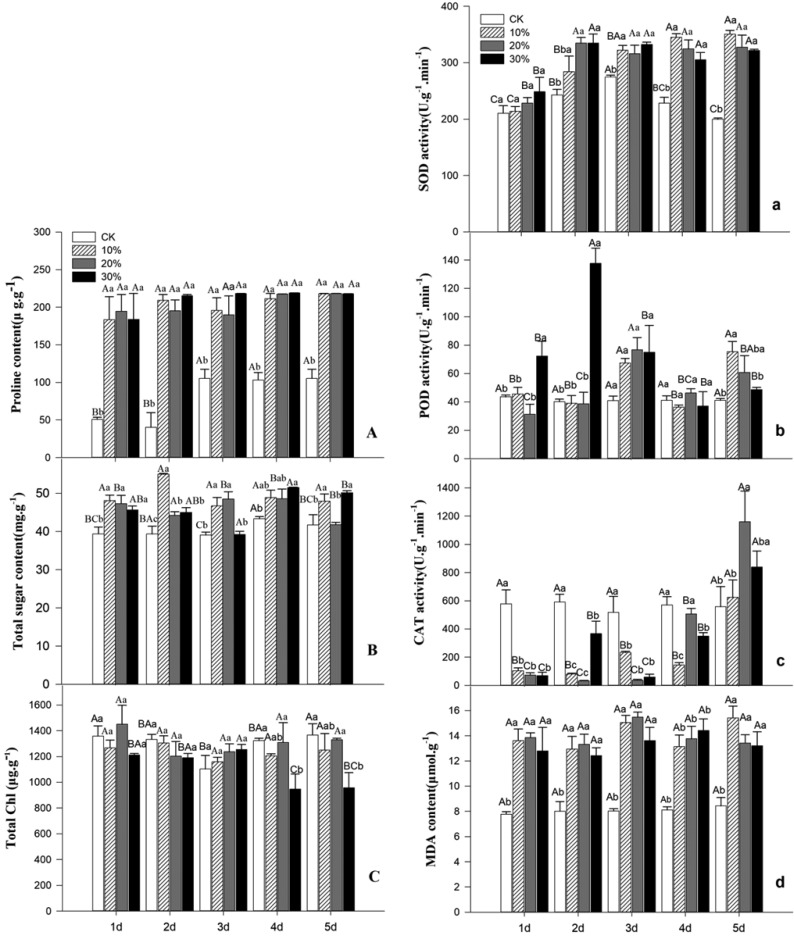
Effects of PEG-mediated drought stress on Physiological index of *Kochia prostrate*. *Note: A B represents the difference of the same treatment group at different times, a b represents the difference between different treatment groups at the same time.

It can be seen from the figure that the chlorophyll content of the *Kochia prostrata* seedlings shows differences due to different stress intensity and stress time. Under the conditions of 10% PEG-6000 concentration (mild stress) and 20% (moderate stress), the total chlorophyll content did not change significantly with the treatment time. Compared with the control, the difference was not significant. Under the condition of 30% concentration (severe stress), the total chlorophyll content decreases with increasing of treatment time. On the 4th and 5th day of treatment, the total chlorophyll content decreased sharply, and the difference was significant compared with the control (Figure 1C).

### 4.2. Osmoregulation substance

Soluble sugars and proline are important osmotic adjustment substances in plants. Under drought stress conditions, the accumulation of osmotic adjustment substances is beneficial to the plants in the leaves of the *Kochia prostrata* to regulate the water balance and adapt to the drought environment.

The content of soluble sugar in the *Kochia prostrata* seedlings increased with the increase of stress intensity and the prolongation of stress time, and most of them were significantly higher than the control (P < 0.05). Among them, under the 10% concentration, the soluble sugar content increased firstly with the increase of time, and the soluble sugar content reached 54.98 mg/g on the second day of treatment, which was 1.14 times of the control group.

Under the condition of 20% drought stress, the soluble sugar content increased first and then decreased with the increase of stress intensity and the stress time. On the fifth day, the soluble sugar content decreased to 41.75 mg/g, which was not significantly different from the control. Under the treatment of 30% drought stress, the soluble sugar content first decreased and then increased (Figure 1B). The content of proline in the seedlings of the *Kochia prostrata* increased rapidly with the increase of stress intensity, and was significantly higher than that of the control (P < 0.05) (Figure 1A). The difference of stress intensity during the same period was not significant.

### 4.3. Antioxidant activities

The SOD activity of the *Kochia prostrata* leaves of the seedlings increased gradually with the prolongation of stress time and the intensity of stress increased two days before treatment. The activity of SOD at 10% stress treatment continued to increase continuously on the third day after treatment, and was in the 5th, the day reaches a maximum of 350.68 U/g min. After 30% stress treatment, the SOD activity of the leaves increased first and then decreased. After the fourth day of treatment, the plants began to wither and the SOD activity decreased. This is its adaptive performance to stress time, in the first two days, 20% and 30% concentration of SOD activity under drought stress was higher than 10% drought stress, and there was no significant difference between 20% and 30% drought stress treatment (Figure 1a).

Under the condition of 10% and 20% stress treatment, the POD activity of the leaves of the *Kochia prostrata* increased with the stress time, and then the fluctuation trend was the same, then the change trend was consistent.

There was no significant difference between the 10% and 20% concentrations of drought stress treatment at the same time. The activity of POD in 30% concentration stress showed a sharp increase and then decreased. On the second day, the maximum value was 137.6 U g^−1^ min, and then gradually decreased (Figure 1b).

The CAT activity of the *Kochia prostrata* leaves decreased first and then increased with the increase of stress time under different stress treatment conditions, and reached the maximum on the 5th day, and the CAT activity on the 5th day under the condition of 20% concentration stress. Up to 1160 U·g^−1^·min, 10% concentration stress treatment except the 5th day, the difference was significant compared with the same control (Figure 1c).

### 4.4. Lipid peroxidation

The content of malondialdehyde in the *Kochia prostrata* seedlings increased with the increase of stress intensity and the prolongation of stress time, and most of them were significantly higher than the control (P < 0.05). With the increase of stress concentration and time, the MDA content increased gradually, and the decrease or increase increased with time. When treated with 20% stress for 3 days, the MDA content reached a maximum of 15.48 µmol/g, which was 192.87% compared with the same control (Figure 1d).

### 4.5. Correlation test between physiological indicators

Correlation analysis of physiological and biochemical indexes of the *Kochia prostrata* leaves under drought stress, There was a negative correlation between chlorophyll content and various physiological indexes, which showed a significant negative correlation with SOD activity. The soluble sugar content was significantly positively correlated with proline content, SOD activity and MDA content, and proline content was significantly positively correlated with MDA content. There was a significant positive correlation between SOD activity and POD activity, and a significant positive correlation with MDA content. CAT activity was significantly negatively correlated with MDA content. The above results indicate that there is a certain correlation between the membrane lipid peroxidation, osmotic adjustment substances, reactive oxygen species and protective enzymes, so that the *Kochia prostrata* can actively cope with drought stress and improve adaptation (Table 1).

**Table 1.** Correlation coefficients of physiological and biochemical indexes in the Kochia prostrata leaves under drought stress.

Item	Total chlorophyll content	Soluble sugar content	Proline content	SOD activity	POD activity	CAT activity
Soluble sugar content	−0.108					
Proline content	−0.215	0.534**				
SOD activity	−0.302*	0.226**	0.652			
POD activity	−0.0352	−0.0195	0.240	0.300*		
CAT activity	−0.0238	−0.247	−0.198	0.0381	−0.00563	
MDA content	−0.189	0.527**	0.713**	0.539**	0.218	−0.263*

*Note: *: P < 0.05; **: P < 0.01.

### 4.6. Effect of drought stress on stomatal

**Figure 2.**
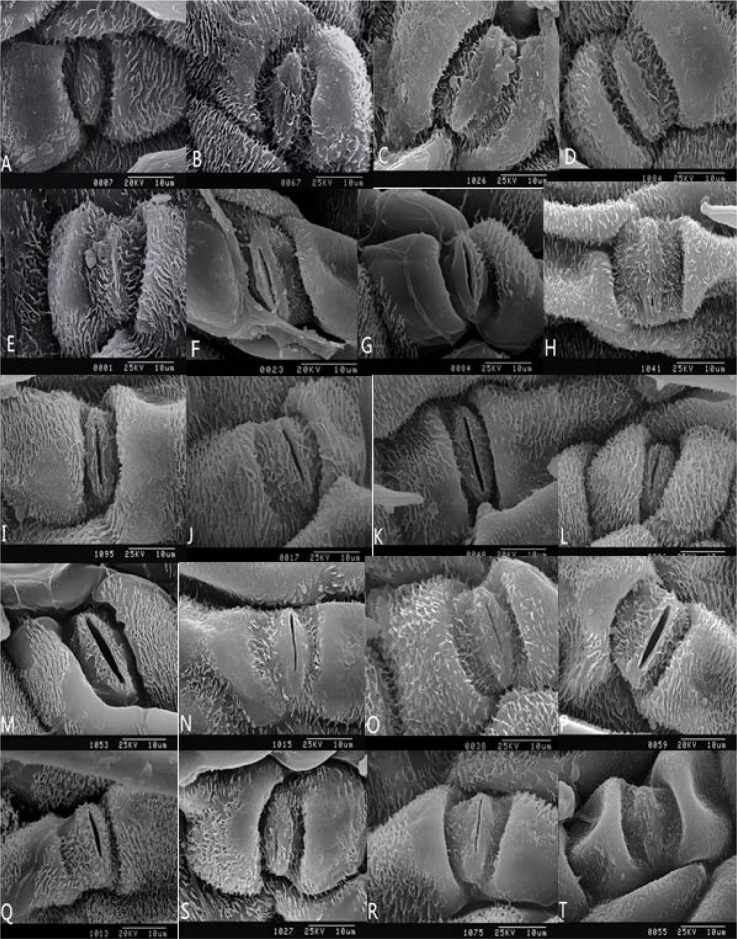
SEM pictures of lower epidermis in leaves of the *Kochia prostrata* under drought stress (3000x). *Note: A–E, F–J, K–O, P–T are stomatal apparatus of the *Kochia prostrata* in control, mild drought, moderate drought, severe drought after 1 to 5 days.

Under the condition of 10% concentration of drought stress, with the prolongation of stress time, the wax covered on the stomatal fell off, the stomatal gradually opened, and some of the stomatals opened more (Figure 2). Under the condition of 20% concentration of drought stress, with the prolongation of stress time, some of the stomatal were opened, and the guard cells collapsed slightly. Under the condition of 30% concentration of drought stress, with the prolongation of stress time, the open stomatals gradually closed and subsided, and the cells around the stomatal contracted and collapsed, and connected to each other to grow strips. Some cells were pleated and folded, and the whole stomatal was completely wrapped together.

**Figure 3.**
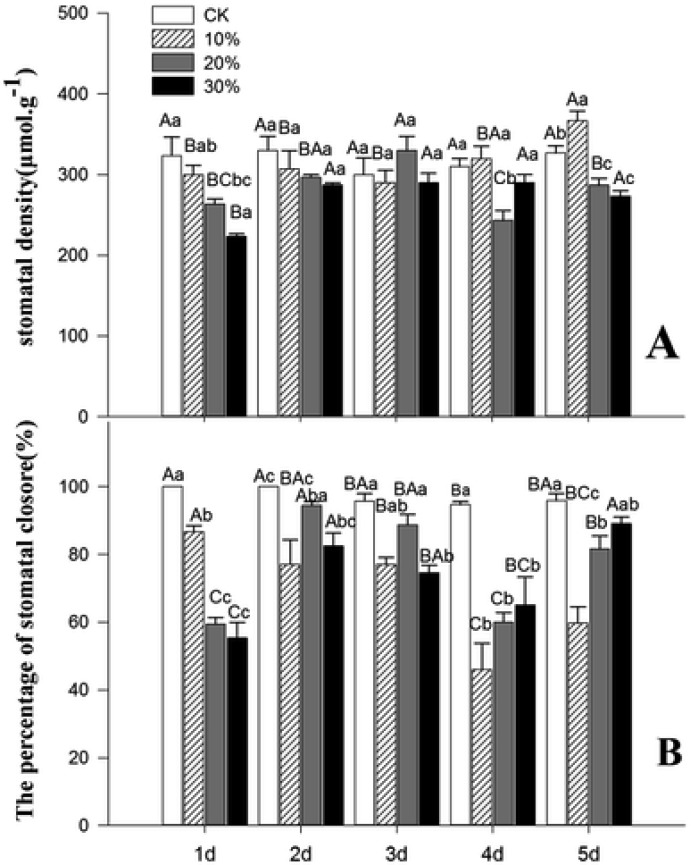
Effects of PEG-mediated drought stress on the stomatal of *Kochia prostrate*. *Note: A B represents the difference of the same treatment group at different times, a b represents the difference between different treatment groups at the same time.

In the first two days of drought stress, with the increase of stress concentration, the stomatal density showed a trend of decreasing. On the 3rd and 4th day of drought stress, the stomatal density showed irregular dynamic changes with the increase of stress concentration. On the 5th day, the stomatal density increased with 10% concentration of drought stress, and the stomatal density reached a maximum of 367. 00 mm^2^ (Figure 3a). The difference was significant compared with the control. As the degree of stress increases, the density of the pores gradually decreases.

Under normal growth conditions, the stomatal of the *Kochia prostrata* are mostly closed, and some of the stomatal gradually open when the drought stress is intensified. Under the condition of 10% concentration of drought stress, the percentage of stomatal closure gradually decreased with the prolongation of time. Under the condition of 20% and 30% drought stress, the percentage of stomatal closure was consistent with the prolongation of time, the trend is to increase first, then decrease and then increase (Figure 3b).

### 4.7. Expression changes of transcription factors in Kochia prostrata leaves under PEG stress

#### 4.7.1. Sequencing data statistics and evaluation

A total of 1,177.46 M Reads were obtained by sequencing, with a total of 352.25 Gbp data and Q30 of 85%. It can be seen from Table 2 that the proportion of clean reads obtained by each group accounts for more than 95% of the original reads, indicating that the quality of the database construction work is good.

#### 4.7.2. Transcription group splicing

The length of the sequence obtained by splicing is used to measure the quality of the splicing. For example, the longer the length of the spliced sequence, the better the sequencing quality. The experimental analysis used denovo assembly, using Trinity software for heavy head assembly, a total of 407,404 “trinity” genes, 754,650 transcripts, and a total of 593 M transcripts. The specific assembly statistics are shown in the Table 2, it can be seen from the data in the table that the spliced fragments have high assembly integrity, the splicing results are long, and the sequencing quality is good.

**Table 2.** Unigene and transcript length statistics.

Item	Unigene	Transcript
Total length (nt)	282,545,522	621,906,093
Total number	407,404	754,650
N50 (bp)	768	1056
Median contig length	493	543
Average contig	693.53	824.10

*Note: N50: The assembled segments are sorted from long to short and the length values are accumulated. The last accumulated fragment length value when the length value is accumulated to 50% of the total length. Unigene: The longest transcript of each gene was used as a representative of the gene, called Unigene, followed by subsequent analysis. nt: The unit of nucleotides.

#### 4.7.3. Gene function annotation

The assembled transcripts were predicted by transdecoder software, and a total of 422,158 protein sequences were predicted. After comparison with the uniport protein database 41,468 proteins was annotated, the unique gene is 24,075.

#### 4.7.4. Gene expression calculation and differential expression gene screening

**Figure 4.**
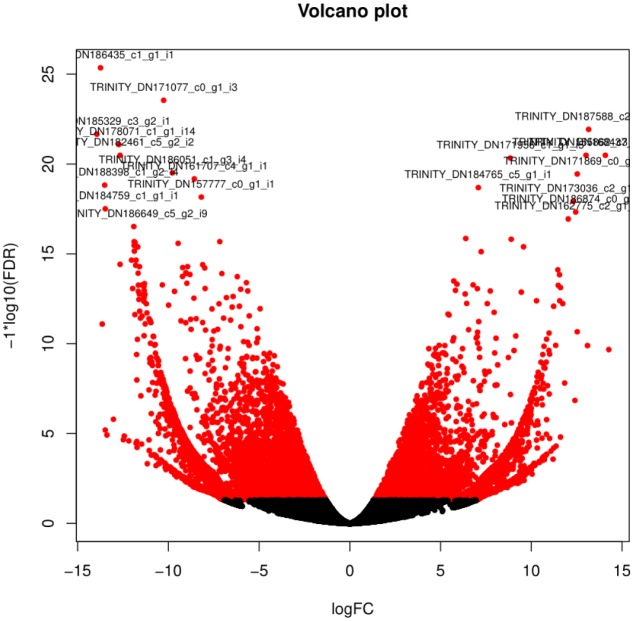
Differential gene screening-CK VS severe drought. *Note: Screening of differentially expressed genes in leaves under control and severe water stress conditions, expressing gene off-group points are the difference.

**Figure 5.**
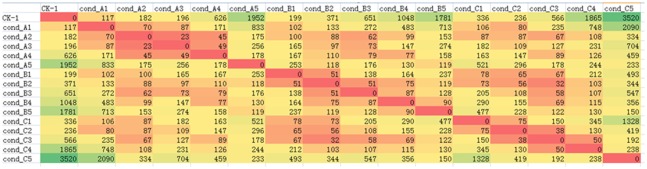
Overview of genetic differences between treatments.

The expression of 754,650 transcripts in 48 leaf samples was calculated by RSEM software, and then the difference gene between the two samples was screened by EdgeR package. A total of 8462 differential genes were obtained between the 16 samples. The statistics of the number of differential genes among different samples are shown in Figure 5. Compared with the control, the number of differential genes increased gradually with the prolongation of stress time, and the number of differential genes on the fifth day of severe stress treatment was the largest, reaching 3,520. Therefore, it is important to comment on GO annotation and KEGG metabolic pathway, and analyze and classify the differential genes. A scatter diagram of all gene expression levels between severe drought stress (C) and control (CK) was drawn (Figure 4), and differentially expressed genes were screened. The screening threshold is P ≤ 0.05, og_2_ Fold Change| > 1, old Change is the ratio of the amount of each gene expressed in the PEG-6000 treated material to the untreated material. Through the scatter diagram, the expression of genes in different treatment expression profiles can be clearly and intuitively seen. According to statistics, there are 2261 up-regulated genes, accounting for 64.23% of the total number of differentially expressed genes, 1259 down-regulated genes, accounting for 35.77%.

#### 4.7.5. Differential gene function analysis

(1). GO analysis of differentially expressed genes.

**Figure 6.**
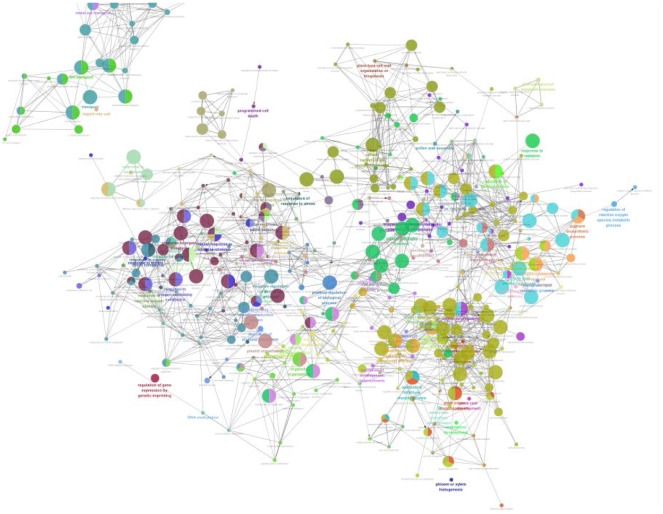
DEG fusion go network. *Note: Differential gene functional enrichment networks (Go biological processes), different colors represent different pathways, and different colors in the same circle indicate the presence of alleles identical by state in two biological processes.

Gene Ontology (GO) is an internationally standardized gene function classification system. GO has a total of 3 ontology, describe the cellular component, molecular function, and biological process of the gene respectively. Functional analysis of differentially expressed genes with significantly enriched GO functions can determine the major biological functions of differentially expressed genes. GO analysis has a suggestive effect on the experimental results. By analyzing the statistical analysis of GO terminology for differentially expressed genes, GO classification entries enriching differential genes can be found, and the most likely related GO terms can be located to find different samples. The differential genes may be related to changes in gene function. Analysis of GO functional analysis of differentially expressed genes between severe drought stress (C) and control (CK) (Figure 6). After the analysis, metabolic process, auxin biosynthetic process, regulation of membrane potential, regulation of intracellular signal transduction, cellular lipid catabolic process, xanthophyll metabolic process, stomatal closure, protein kinase activity and other biological pathways, molecular functions, cell composition, etc. have obvious response changes. In the differential gene annotation to the biological process (BP), a total of 261 Go terms were enriched in the up-regulated gene, and a total of 231 Go terms were enriched in the down-regulated gene, wherein the enrichment was more prominently distributed in the L-amino acid import, carotene metabolic process, trehalose metabolism in response to stress, regulation of auxin biosynthetic process.

(2). Differentially expressed gene KEGG enrichment analysis

**Figure 7.**
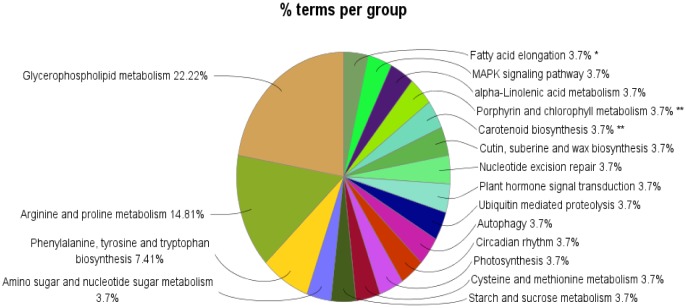
Differentially expressed genes KEGG enrichment analysis.

KEGG is a database for systematic analysis of gene function and genomic information, which can be used for metabolic analysis and metabolic network research. After KEGG metabolic pathway analysis of DEGs between the sample control group (CK) and the severe drought stress group (C), DEGs were obtained in 27 metabolic pathways. Further statistics revealed that the three most prominent Pathway in the 27 KEGG Pathway were Carotenoid biosynthesis, Fatty acid elongation, Porphyrin and chlorophyll metabolism, which mainly involved metabolic pathways, signal transduction and secondary metabolism (Figure 7).

## 5. Discussion

Plants respond and adapt to drought stress by changes in physical structure in leaves and roots, physiology and biochemistry changes, and alterations in gene expressions. These changes can maintain the balance of the synthesis of matter and energy metabolism, and can also improve a plant's ability to survive in an arid environment. Changes in physical structure change include changes stomatal morphology and density, and the physiology and biochemistry changes are mainly characterized by an increase in osmoregulation substance and the synthesis of antioxidants.

Under drought stress, plant display some physiological and biochemical responses to cope with oxidative damage. These responses include active oxygen scavenging system and osmotic adjustment substances.

As the main pigment of plant photosynthesis, chlorophyll plays an important role in the absorption, transmission and conversion of light energy, and its content is closely related to the strength of plant photosynthetic carbon fixation. Plants accelerate when they age under adverse conditions. Under the condition of 30% drought stress, the chlorophyll content of the *Kochia prostrata* decreased sharply on the 4th and 5th day of treatment, and the difference was significant compared with the control. The other treatments showed no significant change, indicating that the *Kochia prostrata* seedlings can adapt to a certain degree of drought and with strong drought resistance. Similarly drought-tolerant genotypes have been reported to maintain higher chlorophyll content than sensitive ones [18].

Pro is an important amino acid in plants, when plants are under drought stress, they can act as penetrating agent for plants and participate in plant osmotic adjustment [19]. The accumulation of Pro has the significance of adapting to drought stress. Under drought stress, Pro elevation can be considered as the physiological response of plants to drought stress. The varieties with poor drought resistance first accumulated Pro, but they showed a downward trend with the extension of stress time, while the varieties with strong drought resistance showed an upward trend under long-term stress. The accumulation of Pro in this experiment remained high levels on the 5th day of drought stress, indicating that the *Kochia prostrata* seedlings have strong drought resistance.

Studies have shown that superoxide dismutase (SOD) activity is an important indicator of plant drought resistance, is one of the important free radical scavengers, is the “first line of defense” against reactive oxygen damage, in the antioxidant system it is at the core. Under the condition of mild drought stress, the activity of SOD reached the maximum on the 5th day of stress, and under the condition of severe drought stress, the activity of SOD reached the maximum on the 2nd day of stress, indicating that the seedlings of the *Kochia prostrata* had strong resistance and the ability to deal with oxygen.

POD is a protective enzyme related to aging in plants. Its role in plants is non-specific. It is related to membrane lipid peroxidation, and it can remove cell bodies to produce less O^2−^, which is resistant to plants. It is closely related to the resilience of plants.

Under 30% concentration of drought stress, POD activity was the same as SOD, reaching a maximum on the second day, and then their activity decreased with time. Studies have shown that mild drought stress can activate the enzyme system in plants and promote the increase of enzyme activity, while severe stress leads to the decrease of the activity of these protective enzymes, and the change is positively correlated with the degree of drought [20].

At the same time, the changes of SOD and POD activities under high concentration of drought stress were higher than those of low concentration drought stress, and reached the maximum on the second day, indicating that the protective enzyme system responded quickly to drought stress. At the late stage of stress, the vitality and balance of the protective enzyme system are destroyed in plants, causing the accumulation of active oxygen, starting and aggravating membrane lipid peroxidation and causing damage to the overall membrane.

CAT can remove H_2_O_2_ from plants, protect aerobic organisms from H_2_O_2_, and maintain ROS at a lower level. It can slow down the accumulation of ROS, reduce the level of membrane lipid peroxidation and other damage processes, and make plants maintain normal growth and development.CAT plays an important role in plant resistance. It indicated that CAT activity was restricted in the early stage of stress, and did not play a major role in the process of scavenging ROS. CAT activity became stronger with the increase of stress intensity and stress time.

When plant tissues are under severe stress, ROS will accumulate in the cells, producing lipid peroxides with strong oxidative properties and degradation products of various small molecules. Among them, the MDA concentration is most significant. Therefore, the concentration of MDA is an important indicator for detecting damage to plant membranes. The MDA content of *Kochia prostrata* increased under different stress concentrations, indicating that the cell membrane system was damaged to varying degrees, but the MDA content did not increase with time, and the MDA content decreased during the process. The treatment level increased first and then decreased with the treatment time, which may be the result of self-protection regulation during stress.

Drought stress not only reduces the photosynthesis of plants, but also changes the tissues, organs and ultrastructure of plants [21]. Stomatal is the gateway for gas exchange between the leaves and the outside. Changes in stomatal opening and density have an important impact on plant water status and CO_2_ assimilation [22]. Stomatal is sensitive to changes in plant habitats. Therefore, plant leaf stomatal parameters (density, size) are mostly used to reflect the response of plants to environmental changes [23].Some studies showed that as the degree of drought increases and the density of stomata increases, some studies have showed that the increase in stomatal density with the increase of drought stress first increases and then decreases. The results showed that the stomatal density increased leaves with the drought stress time under mild drought stress of *Kochia prostrate*, and increased with the prolongation of drought stress under moderate and severe drought stress. The possible reason for the result is the diversity and complexity of the response and adaptation of the leaf stomatal density to environmental conditions.

Drought can lead to dehydration and osmotic stress of plant cells, which seriously affects various normal physiological and metabolic processes in plants. Cells sense drought stress signals and initiate related signal transduction pathways [24], regulates related gene expression and physiological responses while responding to different levels of transcription and translation. Differential expression of biological processes, gene annotation in processes such as regulation of cellular process, cellular catabolic process, signal transduction, organic substance catabolic process, negative regulation of biological process, response to water deprivation, anatomical structure morphogenesis, chloroplast organization, cellular lipid metabolic process, cellular potassium ion transport. This indicates that the drought stress response of the *Kochia prostrata* is a complex physiological process, and it is not a single gene to improve its drought resistance. This study found that the cellular catabolic process (GO: 0044248) up-regulated 56 genes, response to water deprivation (GO: 0009414) up-regulated genes 24, and the organic substance catabolic process (GO: 1901575) up-regulated 56 genes. They are all involved in the response of *Kochia prostrate* to drought stress.

Under drought stress, plants produce endogenous hormones to increase the efficiency of plant water use. In this study, KEGG enrichment analysis of *Kochia prostrata* DEGs revealed that among the significant enrichment differences, Plant hormone signal transduction (KEGG: 04075), Porphyrin and chlorophyll metabolism (KEGG: 00860, Photosynthesis (KEGG: 00195), MAPK signaling pathway (KEGG: 04016) related genes are relatively more. The expression of MAPK signaling pathway (CTR1, EIN4, OST1, RCAR1, SNRK2.4, YDA) was activated after drought stress, thereby finely regulating drought stress signal transduction, RCAR1 was identified as an ABA receptor [25], plants sense water stress and induce the biosynthesis of abscisic acid. ABA can reduce transpiration and resist drought stress by promoting stomatal closure or inhibiting stomatal opening [26]. The mitogen-activated protein kinase (MAPK) is a ubiquitous signal transduction pattern in eukaryotes. The activated MAPK regulates the expression of the stressor gene by phosphorylating downstream transcription factors [27].

In many plants under drought stress conditions, in order to maintain osmotic equilibrium and body water, some small molecular compounds accumulate in the cells, which is beneficial to the water absorption of plants under drought conditions. Under drought stress, proline is mainly composed of glutamate synthesis pathway. In this pathway, Pyrroline-5-carboaylate synthetase (P5CS) is a bifunctional enzyme, which encodes a protein catalytic cracking of glutamate and converts it into Pyrroline-5-carboxylate (P5C). It is a key enzyme encoding gene for proline synthesis. Under drought stress conditions, the expression level of P5CS gene was increased, and the transcription level of PDH was very low. At this time, free proline accumulated in a large amount. It can be seen that the enzyme gene P5CS is the key to controlling the level of proline.

There may be transcription factors involved in the expression of SOD genes in various subcellular structures of plant cells. When the plant is subjected to stress, the specific SOD gene is expressed, and the transcription factor associated with it can quickly leave the organelle and enter the nucleus, and activates or inhibits the transcription of the corresponding SOD gene by binding to the cis-acting element of the SOD gene promoter [28]. At present, transcription factors that regulate the transcription of SOD genes, such as AP2, ACE1, PpSBP2 and GmNAC2, have been reported [29].

Under drought conditions, WRKY gene was overexpressed in tobacco, and compared with wild-type tobacco plants, SOD activity increased, indicating that WRKY transcription factor plays an important role in regulating SOD gene under drought stress [30]. Other studies have shown that AP2, MYB and other transcription factors are also involved in the regulation of SOD genes [31]–[33]. The SOD content increased during the drought stress of *Kochia prostrate*, the gene function analysis of transcription factors indicated that AP2 and MYB were involved in the regulation of *Kochia prostrate* leaf cells under drought stress.

In this study, the physiological and biochemical indexes, stomatal ultrastructural observation and transcriptome analysis of the seedlings of *Kochia prostrata* under drought stress were compared, and the changes of physiological indexes, stomatal state and differentially expressed genes were compared after treatment at different time for further screening, which lays the foundation for studying its drought resistance mechanism.

## 6. Conclusions

Under drought stress conditions, Under the conditions of 10% PEG-6000 concentration (mild stress) and 20% (moderate stress), the total chlorophyll content did not change significantly with the treatment time. Compared with the control, the difference was not significant. The content of soluble sugar in the *Kochia prostrata* seedlings increased with the increase of stress intensity and the prolongation of stress time, and most of them were significantly higher than the control (P < 0.05). The content of proline in the seedlings of the *Kochia prostrata* increased rapidly with the increase of stress intensity, and was significantly higher than that of the control (P < 0.05). The SOD activity of the leaves increased gradually with the prolongation of stress time and the intensity of stress increased two days before treatment. The CAT activity of leaves decreased first and then increased with the increase of stress time under different stress treatment conditions, and reached the maximum on the 5th day. The content of malondialdehyde increased with the increase of stress intensity and the prolongation of stress time, and most of them were significantly higher than the control (P < 0.05). Under normal growth conditions, the stomatal of the *Kochia prostrata* are mostly closed, and some of the stomatal gradually open when the drought stress is intensified. A total of 1,177.46 M Reads were obtained by sequencing, with a total of 352.25 Gbp data and Q30 of 85%. a total of 407,404 “trinity” genes, 754,650 transcripts, and a total of 593 M transcripts. A total of 8462 differential genes were obtained between the 16 samples. According to statistics, there are 2261 up-regulated genes, accounting for 64.23% of the total number of differentially expressed genes, 1259 down-regulated genes, accounting for 35.77%.
